# Synthesis, DFT, Biological and Molecular Docking Analysis of Novel Manganese(II), Iron(III), Cobalt(II), Nickel(II), and Copper(II) Chelate Complexes Ligated by 1-(4-Nitrophenylazo)-2-naphthol

**DOI:** 10.3390/ijms232415614

**Published:** 2022-12-09

**Authors:** Yousef A. A. Alghuwainem, Hany M. Abd El-Lateef, Mai M. Khalaf, Amer A. Amer, Antar A. Abdelhamid, Ahmed A. Alzharani, Anas Alfarsi, Saad Shaaban, Mohamed Gouda, Aly Abdou

**Affiliations:** 1 Department of Veterinary Public Health and Care, College of Veterinary Medicine, King Faisal University, Al-Ahsa 31982, Saudi Arabia; 2 Department of Chemistry, College of Science, King Faisal University, Al-Ahsa 31982, Saudi Arabia; 3 Department of Chemistry, Faculty of Science, Sohag University, Sohag 82534, Egypt; 4 Department of Chemistry, Faculty of Science, Albaha University, Albaha 65528, Saudi Arabia; 5 Chemistry Department, Faculty of Science, Mansoura University, Mansoura 35516, Egypt

**Keywords:** azo ligand, complexes, DFT, docking, antibacterial, antifungal

## Abstract

Novelmanganese(II), iron(III), cobalt(II), nickel(II), and copper(II) chelates were synthesized and studied using elemental analysis (EA), infrared spectroscopy, mass spectrometry, ultraviolet-visible spectroscopy, and conductivity, as well as magnetic measurements and thermogravimetric analysis (TG). The azo-ligand 1-[(4-nitrophenyl)diazenyl]-2-naphthol (HL) chelates to the metal ions via the nitrogen and oxygen centers of the azo group and the hydroxyl, respectively. The amounts of H_2_O present and its precise position were identified by thermal analysis. Density functional theory (DFT) was employed to theoretically elucidate the molecular structures of the ligand and the metal complexes. Furthermore, the quantum chemical parameters were also evaluated. The antimicrobial properties were evaluated against a group of fungal and bacterial microbes. Interestingly, the bioactivity of the complexes is enhanced compared to free ligands. Within this context, the CuL complex manifested the lowest activity, whereas the FeL complex had the greatest. Molecular docking was used to foretell the drugs’ binding affinity for the structure of *Escherichia coli* (PDB ID: 1hnj). Protein-substrate interactions were resolved, and binding energies were accordingly calculated.

## 1. Introduction

Several harmful bacterial and fungal microbes have recently spread worldwide, causing diseases affecting all living organisms. These microbes also affect the food, water, and soil components. Therefore, there is an immediate need to disclose and develop new drug candidates to suppress hazardous microbial development [1,2]. Among the most investigated, metal complexes have gained much concern in pharmaceutical chemistry. Bio-metals, such as Mn, Fe, Co, Ni, and Cu, have an essential role in biochemistry arising from their coordination potential with different biological targets. Moreover, metal coordination with different bio-ligands plays a crucial role in understanding biological processes [3,4]. Within this context, coordination chemistry has shown considerable therapeutic successes in the field of controlling severe ailments, and advancements in various medications have captivated chemists and researchers for decades.

**Scheme 1:**
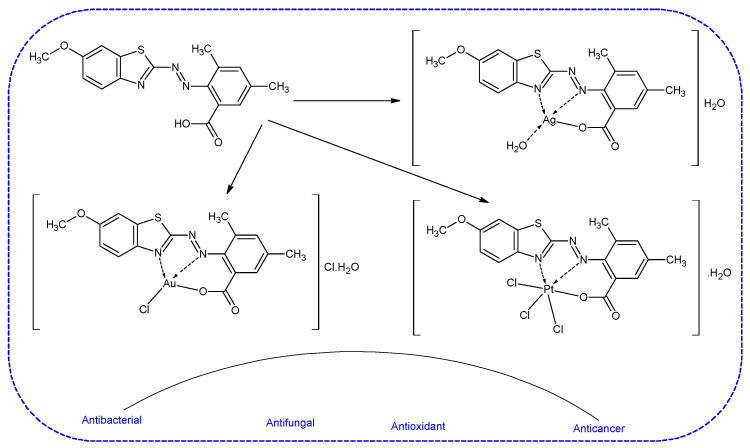
The structures of azo-derived metal complexes with its featuring biological activity (from Ref. [9]).

Azo-dyes are primarily used as a coloring agent, but they also have many other applications in physiochemistry, analysis, bioinorganic, pharmaceutics, and catalysis [5,6]. For example, the sulphonamide-linked azo-dye, Prontosil, is used to treat bacterial infections [7,8] Furthermore, azo-compounds have been the core of concern in drug development owing to their anti-oxidant, anti-viral (HIV), anti-inflammatory, ant-cancer, fungicidal, anti-diabetic, bacteriostatic, and anti-septic activities, Scheme 1 [9,10,11]. Moreover, there has been a great deal of interest in azo-based compounds in both basic and applied research [12]. Moreover, several biological processes linked to azo-based compounds were recently evolved, such as protein synthesis inhibition, DNA and RNA interchelation, and carcinogenesis [13]. In addition, azo-based compounds have received great attention in organic chemistry because of their efficient catalytic activities and their industrial importance in the production of the polymer [14].

In the field of chemical biology, rational design (RD) is an umbrella term that refers to the strategy of creating new molecules with specific functionality. This strategy is based on the ability to predict how the structure will affect the molecule’s behavior when it is simulated using physical models. This is typically performed in conjunction with directed evolution and can be conducted either from scratch or by making calculated modifications to a known structure. In this framework, a rational design was conducted for the preparation of new metal complexes based on a well-known bioactive azo-dye ligand, 1-[(4-nitrophenyl)diazenyl]-2-naphthol (HL). Thus, the goal of this study is to develop new azo-dye-based Co(II), Cu(II), Fe(III), Ni(II), and Mn(II) chelates and assess their bioactivity. In addition, the three-dimensional (3D) structure of the complexes will also be calculated by the DFT computations. Therefore, the current research is expected to provide novel compounds that might be feasible agents for treating differential antimicrobial illnesses.

## 2. Results and Discussion

### 2.1. Characterization of the Structure of the Azo Ligand

The structure of 1-(4-nitrophenylazo)-2-naphthol (HL)was established using IR and NMR spectral data as well as EA. HL showed a broad absorption band of the OH group from 3306-3202 cm^−1^ and aromatic C-H bands at 3057 cm^−1^. Furthermore, the characteristic bands of the N=N group were found at stretching vibration absorption 1550 cm^−1^. The ^1^H-NMR spectra of HL revealed singlet signals at δ 8.44 for the OH group. Also, the aromatic signals were found at δ 8.42−6.71. On the other hand, the ^13^CNMR spectrum showed the following signals: δ 144.59,143.96, 133.04, 131.76, 130.32, 129.90, 128.97, 128.11, 126.53, 126.06, 122.75 and 117.83, Supplementary Materials; Figure S1.

### 2.2. Complexes Structures’ Elucidation

#### 2.2.1. Conductivity and EA Measurements

The produced complexes are H_2_O-insoluble but soluble in DMF and CH_3_CN and stable at 27 °C. Table 1 contains the data on the molar conductivity and EA of the produced compounds. The computed values and the elemental studies of the metal complexes are in accepted agreement with the measured values. The low molar conductivity values showed the non-electrolytic nature of the complexes. 

#### 2.2.2. IR Spectra

The IR was obtained to understand the ligand-metal ion interaction better. The most characteristic IR bands for the ligand and chelates are shown in Table 1. The bands seen originated either from the ligand or the bonds between the metal ion and the coordinating sites of the ligand. The azo (–N=N– the) band was observed at 1550 cm^−1^ in the IR spectrum of the free ligand, whereas the hydroxyl (–OH) band was observed at 3306 cm^−1^.

Comparing the IR bands of the ligand prior to and after chelation with the metal ion, it was found that the bands of the azo (–N=N–) group are still there. However, they were displaced to a lower wave number, 1515–1520, Table 1. This demonstrates that coordination between the metal ion and azo nitrogen took place. It has also been shown that the phenolic (–OH) vibration disappears in all the metal complexes [15]. These results demonstrate the role played by the ligand’s phenolic oxygen in constructing the C–O–M bond during deprotonation. It was previously believed that the –OH groups on H_2_O molecules were responsible for the presence of a broad band in the complexes above 3400 cm^−1^. New spectral bands identified as belonging to the molecules υ (M–O) [16,17] and υ (M–N) [18,19], respectively, Table 1.

These data show that the azo-ligand (HL) forms the complexes via its azo (–N=N–) and phenolic oxygen (–OH) groups, indicating that HL works as a mono-negatively bi-dentate ligand. 

#### 2.2.3. Mass Spectra

Mass spectra are a vital tool for deciphering intricate structures. Therefore, mass spectra were collected to compare the compounds’ relative amounts of various elements and presented in Supplementary Materials; Figure S2. Good concurring was found between the calculated values and the molecular ion peak (M^+^) in the mass spectra of the MnL, FeL, CoL, NiL, and CuL complexes at *m*/*z* 712.46, 748.24, 734.58, 716.13, and 720.88, were in good agreement with 711.5, 747.9, 733.5, 715.3, and 720.14, respectively. Some of the other peaks in the mass spectrum might be attributed to different fragments of metal complexes. The mass spectrum results show a high degree of consistency with the known values for carbon, hydrogen, and nitrogen, as well as with the proposed formula.

#### 2.2.4. Magnetic Moment and Electronic Spectra Measurements

The UV-Vis spectra of ligand and metal complexes were processed in acetonitrile and recorded from 200 to 800 nm, Supplementary Materials, Figure S3 and Table 1. Electronic transitions at 280 and 335 nm in the spectrum of the unbound ligand may be ascribed to π → π* and n → π* states, respectively. These bands are shifted to longer wavelengths after coordinating with a metal ion. One of the best ways to figure out the internal structure of metal complexes is to calculate their effective magnetic moment, μ_eff_ = 2.83 [(X_g_ * Mwt) − (dia magnetic correction * T)]^0.5^], Table 1.

At 395 nm, a band in the electronic spectra of the MnL complex was seen that might be attributed to the ^4^T_2g_ (G) → ^6^A_1g_ transition [20]. The 1.89 B.M. value of the magnetic moment may be explained by the presence of a single unpaired electron with low spin (t_2g_^5^) in an octahedral geometry around the Mn (II) ion.

An electronic spectrum band at 410 nm was seen for the FeL complex, which has been attributed to the ^6^A_1g_ → T_2g_ (G) transition in the complex’s octahedral geometry. The 1.91 B.M. magnetic moment reported for the Fe (III) complex can be explained by the presence of d^5^ low spin (t_2g_^5^) atoms in an octahedral geometry around the Fe(III) center.

A band suggested the octahedral shape in the electronic spectra of the CoL complex at 445 nm, which might be attributed to the ^4^T_1g_ (F) → ^4^T^2g^ (F) transition. The 1.83 B.M. magnetic moment of the CoL complex at room temperature was assigned to the d^7^ low spin (t_2g_^6^ e_g_^1^) electron configuration. Then the CoL complex would have an octahedral shape [21], Table 1.

At 540 nm, bands can be seen in the NiL complex’s electronic spectra that may be attributed to ^3^T_1_ (F) → ^3^T_1_ (P) transitions, suggesting an octahedral geometry around the Ni(II) center, Table 1. NiL complex has a μ_eff_ of 3.14 B.M, which indicates a return to the d^8^ (t_2g_^6^ e_g_^2^) electron configuration [22], Table 1. 

Bands at 525 nm can be attributed to ^2^B_1g_ → ^2^A_1g_ transitions in the electronic spectra of the CuL complex, suggesting an octahedral geometry centered on Cu(II), as shown in Table 1. In addition, the μ_eff_ of the CuL complex was measured to be 1.76 B.M, which is consistent with a return to the d^9^ (t_2g_^6^ e_g_^3^) electron configuration [23], Table 1. 

#### 2.2.5. Stoichiometry of the Metal Complexes

Job’s method of continuous variation was used to calculate the Stoichiometry of the metal complexes [24]. This method is based on the measurement of absorption of a series of solutions in which molar concentrations of two reactants vary, but their sum remains constant. Job’s Method is also known as the method of continuous variation. The principle of the method is that the mole ratio of the metal ion and the ligand is varied between 0 and 1 at constant total concentration C = C_ligand_ + C_metal_. Then the absorbance of each mixture was obtained after allowing the reaction mixtures (M & L) to equilibrate. As a result, the absorbance of each solution was plotted against the ligand mole fraction ([L]/[L]  +  [M]). So, on drawing the graph between the absorbance of the prepared series of solution and their corresponding mole fraction, the exact ratio of metal and ligand at equilibrium can be observed at the maxima of the graph. Maximum absorbance, as measured by the curve of continuous change, occurred at a ligand mole fraction ([L]/([L] + [M])) of 0.66, suggesting the complex construction at a 1:2 (M:L) molar ratio, Figure S4.

#### 2.2.6. Thermal Decomposition

**Figure 1:**
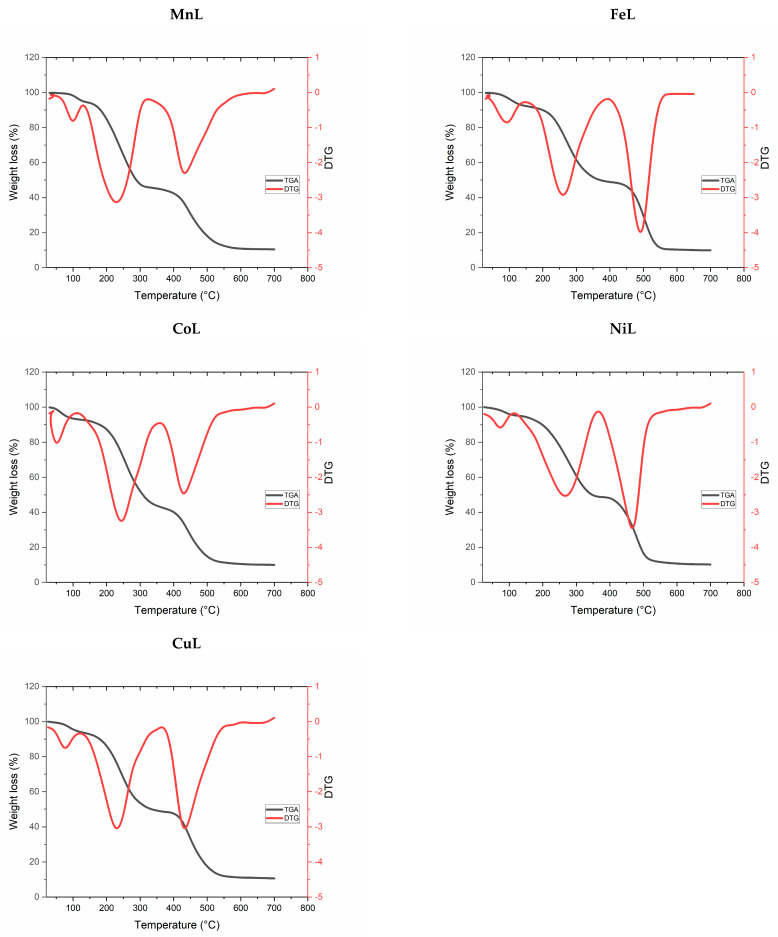
Thermal decomposition of the synthesized complexes.

Determining the relative amounts of coordinated and uncoordinated H_2_O molecules in the examined metal complexes is a crucial step toward understanding their structural features after being subjected to heat degradation [25,26,27], Figure 1 and Table 2.

The first degradation step was found (calc.) weight loss percentage of 5.31 (5.26), 7.24 (7.35), 7.22 (7.37), 5.18 (5.04), and 5.11 (5.08), which linked to the elimination of two, three, three, two and two H_2_O of hydration for the MnL, FeL, CoL, NiL and CuL complexes, respectively, Figure 1 and Table 2.

The second degradation step was observed at found (calc.) weight loss percentage of 49.15 (49.37), 44.31 (44.48), 48.87 (48.98), 46.73 (46.85), and 46.08 (46.14) %, which correlated to the elimination of C_18_H_15_N_4_O_4_, C_16_H_14_N_3_O_3_Cl, C_20_H_15_N_4_O_3_, C_18_H_13_N_3_O_4_, and C_19_H_14_N_3_O_3_ in the case of MnL, FeL, CoL, NiL and CuL complexes, respectively, Figure 1 and Table 2.

The decomposition was continued for the third degradation step to eliminate the remaining organic moiety leaving the metal oxide as metallic residue, Figure 1 and Table 2.

### 2.3. DFT Calculations

**Figure 2:**
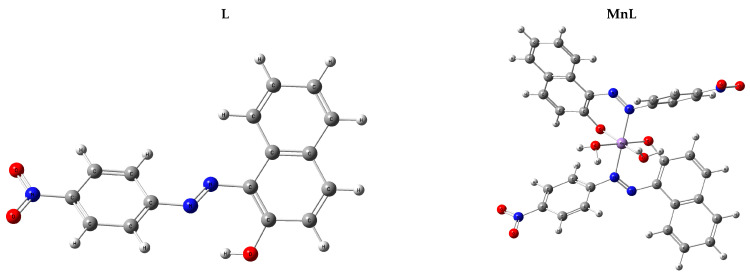

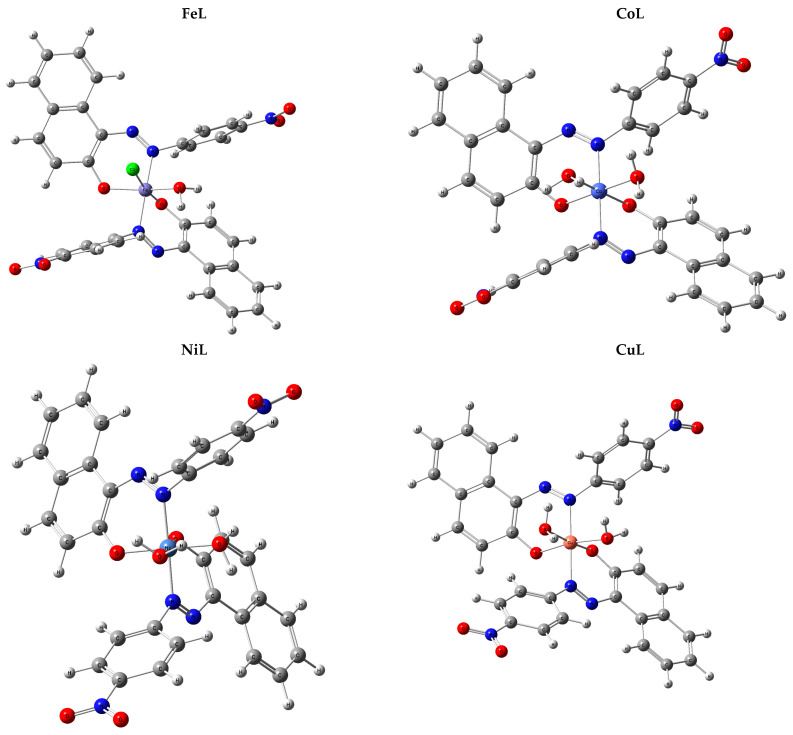
3D optimized structure of the compounds.

Titled compounds that were optimized had an octahedral geometry around the metal core, as; [Fe (L)_2_ (H_2_O) (Cl)] for FeL, and [M (L)_2_ (H_2_O)_2_], for MnL (M=Mn), CoL (M=Co), NiL (M=Ni) and CuL (M=Cu), Figure 2.

**Figure 3:**
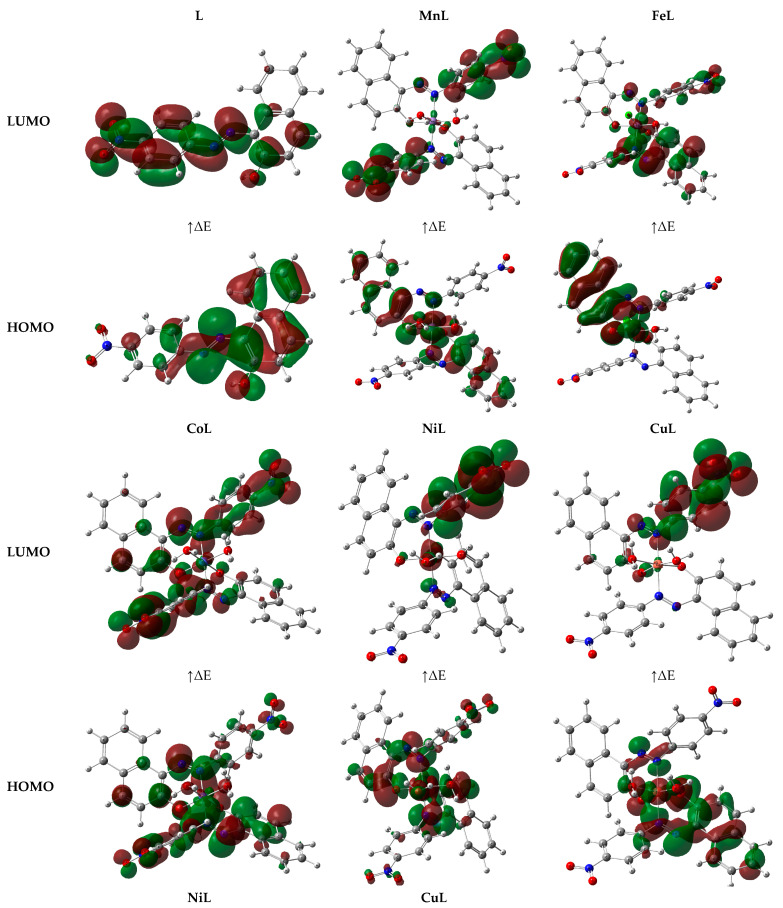
HOMO—LUMO of the optimized structures.

The HOMO and LUMO are two examples of frontier molecular orbitals (FMOs), which have a profound impact on reactivity, chemical stability, and electronic properties (LUMO). A diagram depicting the HOMO-LUMO ratio of the compounds mentioned above is presented in Figure 3. This means that the electrons are dispersed throughout the molecule. Therefore, HOMO-LUMO energies are used to determine different chemical properties such as the ΔE, IP, EA, χ, μ, η, σ, ω, and Nu Table 3.

The HOMO-LUMO energies are many-sided because of their potential application to understanding molecules’ chemical reactivity, kinetic stability, hardness-softness, biological properties, and polarizability. The HOMO was the most distant electron-containing orbital and hence the most generous giver of electrons. The lowest unoccupied molecular orbital (LUMO) functioned as an electron acceptor. Since the HOMO and LUMO orbitals define the molecule’s stability, they also define its susceptibility to attack by nucleophiles and electrophiles [28]. The difference between E_LUMO_ and E_HOMO_ energy levels (ΔE gap) indicates how reactive a molecule is [29,30,31,32]. To that end, molecules with smaller ΔE are more receptive to docking (CuL > MnL > NiL > FeL > CoL > HL).

The chemical reactivity scale also considers how hard or soft an element is. To describe how likely a molecule is to connect with another, the hard-soft-acid-base (HSAB) rule can be employed. The Hard Soft Acid Base (HSAB) rule states that hard acids prefer to connect with other hard acids and bases, while soft acids prefer to interact with other soft acids and bases. Biological macromolecules such as proteins and cells are examples of soft molecules [1,2]. When it comes to dealing with biological molecules, it is preferable to use soft molecules rather than rigid ones. As a consequence of this, greater levels of biological activity are linked to surfaces that are both more soft and less hard. Thus, chemical reactivity follows that CuL > MnL > NiL > FeL > CoL > HL. Table 3 shows a rise in biological activity when the values of softness and hardness increase and decrease, respectively.

The negative chemical potential value [29] demonstrates the stability of the cited compounds. It has a high electrophilicity index and low chemical potential value, both of which are conducive to its electrophilic activity [30]. Both the substrate and the protein contain partial charges, and these charges have a considerable influence on how rapidly they connect to one another. The molecular electrostatic potential (MEP) diagram may be used to gain insight into the 3D architecture and topology of substrates. The MEP determines where the nuclei or electrons impact the molecular geometry most.

**Figure 4:**
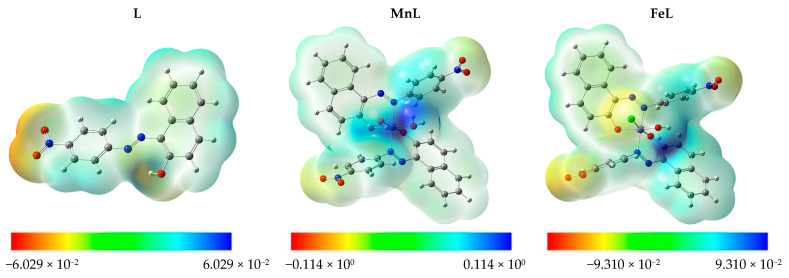

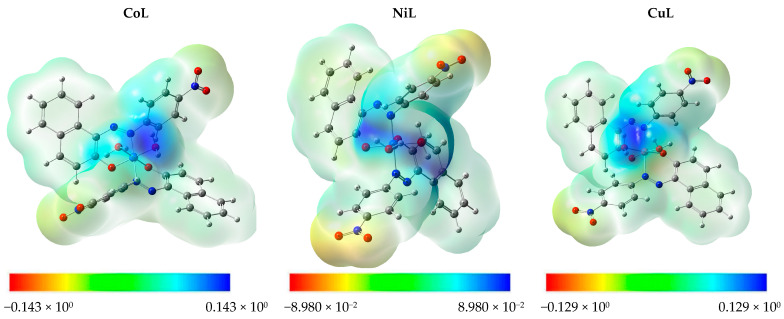
MEP of the compounds.

Each value in an MEP diagram represents a distinct color, from blue to red. The MEP’s positive (blue) and negative (red) regions are linked to nucleophilic and electrophilic reactivity, respectively. Colors in the red spectrum represent portions of the surface that are negatively charged (i.e., the most favorable areas are accepting an electrophile). The attraction of the compound’s suitable sites in interactions with electrophiles is represented by a decrease in the compound’s negative charge [31]. The theoretical map of the MEP diagram for the specified compounds is shown in Figure 4. Negative areas (red) are concentrated around hetero atoms (O and N) in the studied substrates because of the increased electron abundance in these locations. Electrophilic assault is especially effective in these areas. More positive regions (blue) include a coordinated water moiety and metal center, which may function as H-bond donors in protein-substrate intermolecular interactions, Figure 4.

### 2.4. In Vitro Antimicrobial Activity

The potential antimicrobial applications of the compounds were investigated by testing against *P. aeruginosa*, *E. coli* (−ve), *S. aureus* (+ve), and *B. cereus* (+ve) bacterial strains and against *A. flavus*, *T. rubrum*, and *C. albicans* fungal strains using the agar diffusion assay Table 4.

The newly prepared complexes showed more significant biological activity with higher IZ and lowered MIC than the free ligand. Within this context, the MnL and CuL complexes showed promising antibacterial activities with high % Activity Index of 94.44%, 94.44% against *P. aeruginosa*, 90.00%, 90.00% against *E. coli*, 88.89%, 88.89% against S. aureus, and 88.89%, 94.44% against *B. cereus*. Similarly, MnL and CuL complexes showed promising antifungal activities with % Activity Index of 89.47%, 89.47% against *A. flavus*, 81.82%, 81.82% against *T. rubrum*, and 80.95%, 85.71% against *C. albicans.*

The minimum inhibitory concentration, abbreviated as MIC, is typically the point of departure for larger preclinical assessments of potentially novel antimicrobial treatments. The minimum inhibitory concentration (MIC) was found by subjecting a sample to a series of dilutions. The lowest compound concentration that inhibits the growth of bacteria/fungi was recorded as the MIC. MICs are normally reported in ppm. In this framework, the MIC was determined for the tested compounds and listed in Table 4. The metal complexes showed lower MIC (25–6.25 ppm) than the free ligand (around 50 ppm), Table 4. 

The chelation theory interprets such superior activities [32,33,34]. Within this context, complexation diminishes the metal ions’ polarity via the distribution of their positive charges to the neighbor’s donor atoms. This may lead to electron-delocalization throughout the phenyl ring. Because of this result, the complex is more lipophilic, which increases the likelihood that it will be able to penetrate the lipid bilayer of the cell membrane. Furthermore, the complex may affect microorganisms’ metabolic pathways and respiration by interfering with their binding sites. This stops bacteria from making proteins, stunting their growth and eventually killing them.

It was shown that the activity of the complexes increased with the strength of the bond between the metal and ligand, the size of the cation, the number of receptor sites, the rate of diffusion, and the combined action of the metal and ligand to inactivate the biomolecules [35]. In addition, the IZ values for the specified compounds were compared to those of the antibiotic Chloramphenicol, which indicates a high activity index (%), Table 4.

The biological activity against the *E. coli* (G−) of the currently studied complexes was compared to that of previously reported metal complexes from the literature survey [1,2,3,4,36,37,38], Supplementary Materials, Table S1. This compression indicated the high biological activity of the current complexes.

### 2.5. Molecular Docking

The complexes were docked to the dedicated antimicrobial target protein to verify the relationship between antimicrobial results and the inhibitor’s binding affinities. The highest binding affinities may be predicted by molecular docking studies, which use virtual compound screening and scoring systems to do so. This strategy examines the three-dimensional jigsaw puzzle-like fit between two molecules, such as a substrate and the active site binding of the target receptor.

**Figure 5:**
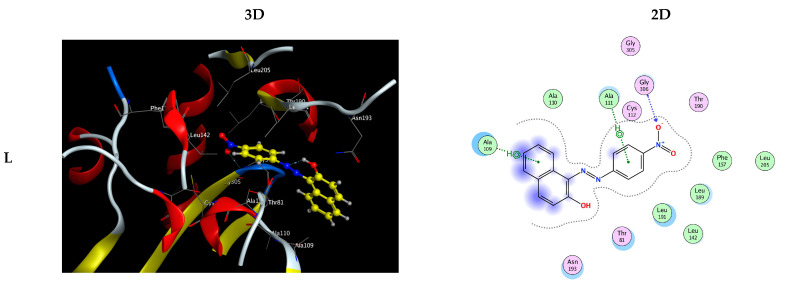

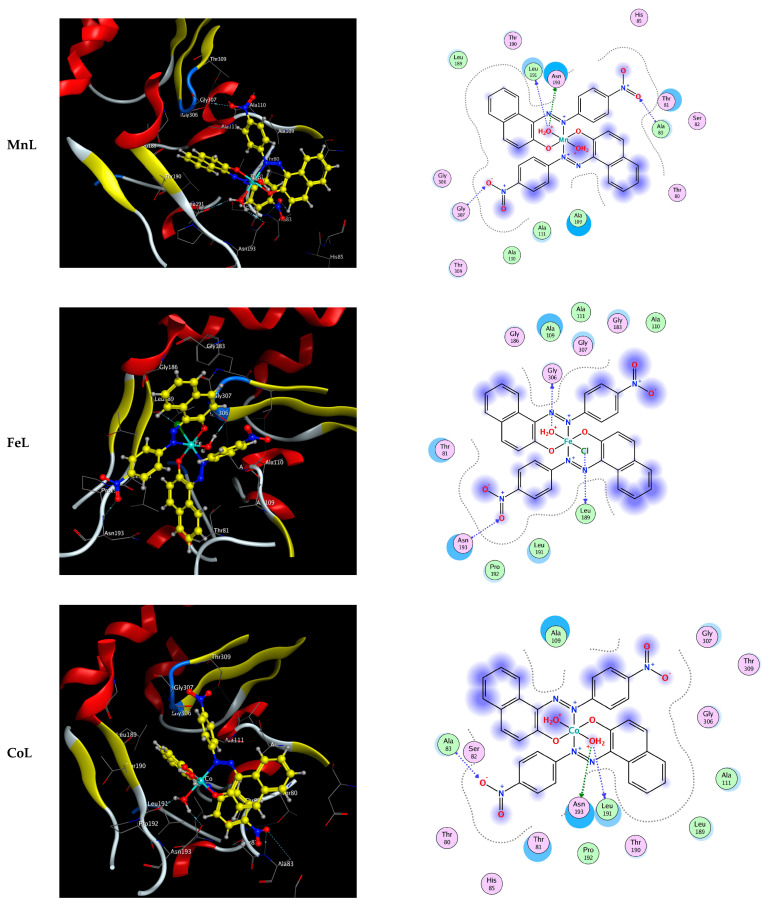

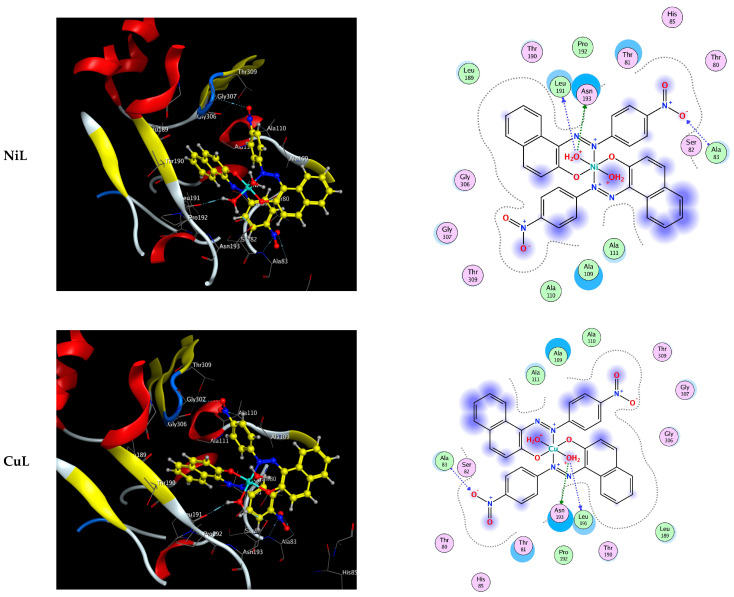
3D orientation of the substrate-protein complex.

Molecular docking of the titled compounds was studied to study their potential against 1hnj. First of all, there is a need to understand why this PDB was chosen and downloaded from https://www.rcsb.org/ (accessed on 1 June 2022). 1HNJ is for the *E. coli* FabH-CoA complex. FabH receptors are considered to be targeted to know the potential of molecules as antimicrobial in nature. FabH is involved in the biosynthesis of fatty acids [35,39]. *E. coli* (PDB ID: 1hnj) is the target receptor, while the aforementioned chemicals comprise the substrate. Both Table 5 and Figure 5 display the results of the molecular docking analysis. The optimum substrate conformation within the binding pocket is depicted in Figure 5.

Particularly intriguing is the fact that, upon docking to the 1hnj pocket (S), the relevant substrates display both robust hydrophobic contacts and a plethora of hydrogen bonds, Figure 5 and Table 5. This demonstrates that docked substrates make strong contacts with the receptor’s active site. The most inhibitory HL were CuL and MnL, followed by NiL, CoL, and FeL. Figure 5 and Table 5 show that the most active molecules, CuL and MnL, were docked to the substrate binding pocket of 1hnj thanks to a combination of hydrogen bonds and hydrophobic interactions.

## 3. Materials and Methods

All chemicals used were analytical reagent (AR) grade, the purest form commercially available. One example is 1-(4-nitrophenylazo)-2-naphthol, but others include MnCl_2_, FeCl_3_, CoCl_2_, NiCl_2_, and CuCl_2_. In addition, both absolute EtOH and CH_3_CN were available from BDH as spectroscopically pure organic solvents.

### 3.1. Synthesis of the 1-(4-Nitrophenylazo)-2-naphthol as Azo-Ligand (HL)

#### 3.1.1. Step I: The Synthesis of Diazonium Salt of 4-Nitroaniline as Azo Compounds

In a round-bottom flask with a capacity of 100 milliliters and a magnetic stirring bar, 1.38 g (10 mmol) of 4-nitroaniline and 36.0 milliliters of concentrated hydrochloric acid were mixed. A progressive addition of 0.69 g (10 millimoles) of sodium nitrite that had been dissolved in 20 milliliters of water was carried out while ensuring that the temperature of the solution remained below 5 degrees Celsius. Keep the diazonium salt solution in an airtight container and store it in the refrigerator. When tested on starch iodide paper, diazonium salt generated a color that may be described as blue-black. The diazonium salt coupling operation was started as soon as it was feasible for us to do so., Scheme 1.

#### 3.1.2. Step II: Coupling Procedure

**Scheme 2:**
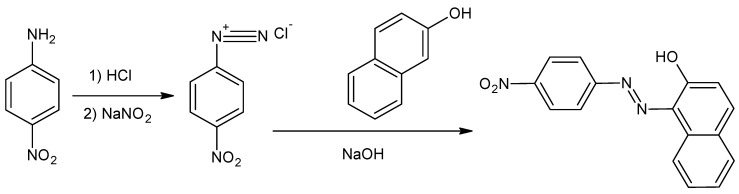
The procedures involved in the production of the free ligand.

The 2-naphthol (1.44 g, 10 mmol) was put into a 50 mL round-bottom flask, and then NaOH (10% *w*/*v*) was poured into the flask. After that, the flask was placed in an ice bath (0–5 °C). After being agitated at zero degrees Celsius to five degrees Celsius for an hour, the cooled diazonium salt solution was added gradually. Then, after filtering the crude residue, washing it many times in cold water, and recrystallizing it from the appropriate solvent, the final azo product was achieved, Scheme 2.

1-(4-nitrophenylazo)-2-naphthol (HL); red powder; Yield (97%); mp. 248–250 °C; IR (cm^−1^): 3306-3202 (–OH), 3057 (Ar-H), 1550 (N=N); ^1^H-NMR (400 MHz, DMSO-d_6_) δ (ppm): 8.44 (s, 1H, –OH), 8.42−6.71 (m, 10 H, Ar-H); ^13^C-NMR (100 MHz, DMSO-d_6_) δ (ppm): 144.59,143.96, 133.04, 131.76, 130.32, 129.90, 128.97, 128.11, 126.53, 126.06, 122.75, 117.83; EA for C_16_H_11_N_3_O_3_ (Calcd./Found); C, 65.53/65.41; H, 3.78/3.61; N, 14.33/14.21.

### 3.2. Metal Chelates Preparation

**Scheme 3:**
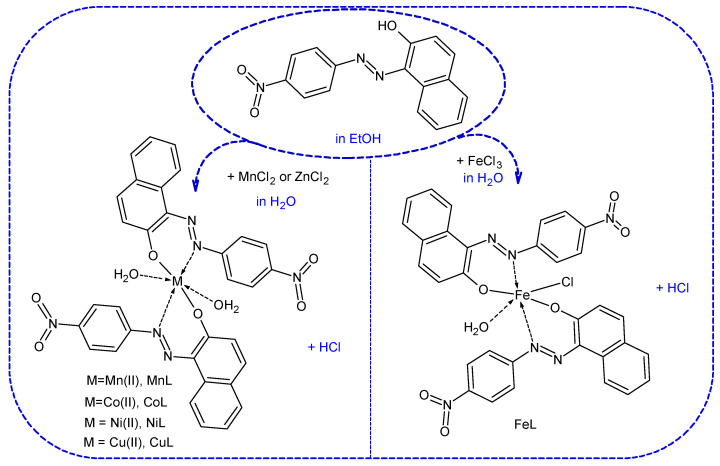
Synthesis of the metal complexes.

The ligand (2.0 mmol) was dissolved in EtOH and heated, and then a solution of the metal salt (2.0 mmol) in H_2_O (20 mL) was slowly added. The furnished mixture was stirred continuously while refluxed in an 80 °C H_2_O bath for 12 h. After being filtered, dried, and washed with the H_2_O−EtOH (1:2) combination, the final product was recrystallized, Scheme 3. Each product’s yield and melting or decomposition temperature were tabulated in Table 1.

### 3.3. Characterization

The chelates composition was determined by IR, molar conductance (of 10^−3^ mol/L ethanol solution), thermal degradation (TGA), electronic spectrum (of 10^−3^ mol/L acetonitrile solution), magnetic measurements, and EA (C, H, and N content). The latter was run on a [Perkin-Elmer 2408] analyzer in the Central Laboratory at the University of Cairo. The infrared spectra were acquired by observing KBr in a Shimadzu DR-8001 spectrometer. The ^1^H-NMR was measured using Bruker DRX (400 MHz) (USA) using TMS (standard) and DMSO-d6. UV-Vis spectra were detected using a Jenway spectrophotometer. We used a Shimadzu type 60 H analyzer to conduct thermal decomposition (TGA) on the named compounds. The molar conductance was measured using JENWAY instrument (model 4320) and a 10^−3^ mol/L EtOH solution. The molar magnetic susceptibility of powdered materials was quantified employing a Bartington Susceptibility apparatus, model 4320. Finally, the compounds’ stoichiometries were determined using the continuous-variation spectrophotometric jobs technique [37].

### 3.4. DFT Calculations

With the use of the 6-311 (d, p) and LANL2DZ basis sets in conjunction with the hybrid correlation functional (B3LYP) [39,40], for the ligand and its complexes, respectively, geometry optimizations of the subject ligand and its complexes were performed [1,2,3,4]. Quantum chemical properties were determined by using the values of the lowest unoccupied molecular orbital (LUMO) and highest occupied molecular orbital (HOMO) energies of the compounds in question, as follows: chemical potential (µ = −χ), electronegativity (χ = (IP + EA)/2), energy gap (ΔE = E_LOMO_ − E_HOMO_), chemical hardness (η = (IP − EA)/2), nucleophilicity index (N = 1/ω), softness (σ = ½ η), maximum electronic charge (ΔN_max_ = −μ/η), and electrophilicity index (ω = µ^2^/2 η) where EA and IP, are electron affinity (EA = −E_LUMO_) and ionization potential (IP = −E_HOMO_) [21,22,28,31].

### 3.5. Antimicrobial Exploration

The antimicrobial activity of the titled compounds was evaluated using a panel of bacteria (*Pseudomonas aeruginosa* (*P. aeruginosa*) (−ve), *Escherichia coli* (*E. coli*) (−ve), *Staphylococcus aureus* (*S. aureus*) (+ve), and *Bacillus cereus* (*B. cereus*) (+ve)) and fungi (*Aspergillus flavus* (*A. flavus*), *Trichophyton rubrum* (*T. rubrum*), and *Candida albicans* (*C. albicans*)). The good agar diffusion technique tested antibacterial and antifungal activities [15,21,23,28]. The findings were monitored using the diameter (in mm) of the inhibitory zone (IZ). 

We found the widely used antimicrobial Chloramphenicol effective against bacteria and fungi using the same method. The % Activity Index, defined as (IZ of test compound/IZ of Standard) × 100, was used to compare the anti-microbial efficacy of the identified compounds to that of the reference [1,2,3,4,22,31].

The minimal inhibitory concentration (MIC) values were determined by serial dilution method using the same above protocol. The tested compounds with different concentrations of 100 ppm, 50 ppm, 25 ppm, 12.50 ppm, and 6.25 ppm were used. The lowest compound concentration that inhibits the growth of bacteria/fungi was recorded as the MIC.

### 3.6. Molecular Docking

Through the MOE software, molecular docking against *E. coli* (PDB ID: 1hnj) confirmed the therapeutic efficacy of the reported drugs [40,41,42,43].

The 3D structure of the target protein receptor was retrieved from the protein database at http://www.rcsb.org (accessed on 1 June 2022). The compounds of interest were used as a substrate. In this molecular docking evaluation, we use the MOE software, which simulates a molecular smorgasbord.

After establishing a new database for each compound in MDB format, we optimized them for substrate preparation by reducing their energy demands. Hydrogen atom addition, receptor type connections, potential energy fixation, active pocket search, and dummy generating are all steps in the receptor preparation process. Docking patterns and interaction parameters were exported to score inhibitory activity using a scoring function (S, kcal/mol) and analyze interaction features [22,23,28,31].

## 4. Conclusions

Five novels of CuLQ, CoL, FeL, MnL, and NiL complexes were synthesized and analyzed using physicochemical and spectroscopic techniques. The data showed that the HL ligand binds to the metals in a 1:2 molar ratio, exhibiting the behavior of a monobasic bi-dentate NO ligand. Spectroscopic measurements showed an octahedral shape for the complexes. Furthermore, their antimicrobial activities were evaluated against various bacterial and fungal pathogenic strains. Moreover, the quantum chemical parameters were determined after theoretically optimizing the molecular structures of the metal complexes. The metal complexes showed more promising antibacterial candidates than the free ligand. In addition, molecular docking was used to determine whether the studied chemicals inhibited *E. coli* growth (PDB ID: 1hnj). The binding of the CuL complex to the target receptor was the most impressive among these drugs.

## Figures and Tables

**Table 1:** EA, conductivity, Uv-vis, and FT-IR results.

		L	MnL	FeL	CoL	NiL	CuL
**Physical properties**	Color	Orange	Pale yellow	Darck violet	Darck orange	Orange red	Pale pink
	Melting point (°C)	190	280	268	255	295	290
	Yield (%)	80	88	85	90	95	90
**EA** **Found (calc.) %**	**C**	65.41 (65.53)	54.27 (54.02)	51.64 (51.39)	52.13 (52.40)	53.48 (53.73)	53.82 (53.37)
	**H**	3.61 (3.78)	3.55 (3.97)	3.92 (3.77)	4.01 (4.12)	4.15 (3.95)	3.27 (3.92)
	**N**	14.21 (14.33)	11.43 (11.81)	11.04 (11.24)	11.18 (11.46)	11.98 (11.75)	11.35 (11.67)
	**M**	**-----**	7.54 (7.72)	7.81 (7.47)	7.88 (8.03)	8.06 (8.21)	8.44 (8.82)
**conductivity**	**µ_v_, Ω^−1^ cm^2^ mol^−1^**	------	8.57	10.14	9.85	9.10	10.08
**IR spectra**	**υ (–OH)**	3306	3457	3460	3455	3448	3466
	**υ (–N=N)**	1550	1515	1520	1518	1520	1517
	**υ (M–O)**	-------	561	558	559	560	557
	**υ (M–N)**	-------	453	451	450	454	455
**UV-vis.**	**λ_max_, nm**	280, 335	395	410	445	540	525
**Magnetic**	**µ_eff_ (B.M)**	-------	1.89	1.91	1.83	3.14	1.76
**Stoichiometry**	**M:L**	-------	1:2	1:2	1:2	1:2	1:2

**Table 2:** Thermal decomposition data.

	TG (°C)	DTG (°C)	Mass Loss (%)		Assignment	Residue
			Found	Calculated		
**MnL**	30–140	95	5.31	5.26	2 H_2_O	MnO
	140–315	225	49.15	49.37	C_18_H_15_N_4_O_4_	
	315–560	430	35.22	35.39	C_14_H_7_N_2_O_3_	
**FeL**	30–140	90	7.24	7.35	3 H_2_O	½ Fe_2_O_3_
	140–360	260	44.31	44.48	C_16_H_14_N_3_O_3_Cl	
	360–550	490	36.44	36.51	C_16_H_6_N_3_O_2_	
**CoL**	30–125	60	7.22	7.37	3 H_2_O	CoO
	125–350	350	48.87	48.98	C_20_H_15_N_4_O_3_	
	350–535	530	33.65	33.42	C_12_H_9_N_2_O_4_	
**NiL**	30–130	75	5.18	5.04	2 H_2_O	NiO
	30–130	70	46.73	46.85	C_18_H_13_N_3_O_4_	
	130–360	260	37.76	37.62	C_14_H_11_N_3_O_3_	
**CuL**	360 520	470	5.11	5.08	2 H_2_O	CuO
	130–335	230	46.08	46.14	C_19_H_14_N_3_O_3_	
	335–540	440	37.84	37.78	C_13_H_10_N_3_O_4_	

**Table 3:** Calculated DFT parameters of the subject compounds.

	*E_HOMO_*	*E_LUMO_*	*∆E*	*I*	*A*	*χ*	*CP*	*η*	*σ*	*ω*	*Nu*	µ
**L**	−6.11	−3.24	2.87	6.11	3.24	4.67	−4.67	1.43	0.35	7.61	0.13	3.26
**MnL**	−4.69	−3.48	1.21	4.69	3.48	4.08	−4.08	0.60	0.83	13.83	0.07	6.77
**FeL**	−6.13	−3.60	2.53	6.13	3.60	4.86	−4.86	1.26	0.40	9.35	0.11	3.85
**CoL**	−6.16	−3.48	2.68	6.16	3.48	4.82	−4.82	1.34	0.37	8.65	0.12	3.59
**NiL**	−5.35	−3.60	1.75	5.35	3.60	4.47	−4.47	0.87	0.57	11.44	0.09	5.11
**CuL**	−4.09	−3.39	0.70	4.09	3.39	3.74	−3.74	0.35	1.43	20.00	0.05	10.70

**Table 4:** The antimicrobial properties of the compounds in terms of zone inhibition (IZ, mm), and activity index (%).

Compounds	*Pseudomonas aeruginosa*			*Escherichia coli*			*Staphylococcus aureus*			*Bacillus cereus*			*Aspergillus flavus*			*Trichophyton rubrum*			*Candida albicans*		
	IZD (mm) ^a^			A%			MIC			IZD (mm) ^a^			A%			MIC			IZD (mm) ^a^		
	8	44.44	50	8	40.00	50	7	A%	MIC	IZD (mm) ^a^	A%	MIC	IZD (mm) ^a^	A%	MIC	IZD (mm) ^a^	A%	MIC	IZD (mm) ^a^	A%	MIC
**L**	17	94.44	6.25	18	90.00	12.5	16	38.89	50	8	44.44	50	8	42.11	50	9	40.91	50	8	38.10	50
**MnL**	15	83.33	12.5	17	85.00	12.5	15	88.89	12.5	16	88.89	12.5	17	89.47	25	18	81.82	12.5	17	80.95	25
**FeL**	16	88.89	12.5	17	85.00	25	15	83.33	25	15	83.33	25	16	84.21	12.5	17	77.27	12.5	17	80.95	25
**CoL**	16	88.89	25	18	90.00	12.5	15	83.33	12.5	16	88.89	25	16	84.21	25	16	72.73	25	18	85.71	12.5
**NiL**	17	94.44	12.5	18	90.00	25	16	83.33	25	16	88.89	12.5	16	84.21	25	17	77.27	25	17	80.95	25
**CuL**	17	94.44	12.5	18	90.00	25	16	88.89	25	17	94.44	6.25	17	89.47	12.5	18	81.82	12.5	18	85.71	12.5

^a^ Inhibition zone diameters (mm) determined using six mm disks were soaked with 20 μM of the compounds.

**Table 5:** Molecular docking data.

	Ligand	Receptor	Interaction	Distance	E (kcal/mol)	S (kcal/mol)
**L**	O 20	GLY 306	H-acceptor	3.33	−1.30	−6.79
	6-ring	ALA 109	pi-H	3.57	−0.70	
	6-ring	ALA 111	pi-H	3.87	−0.90	
**MnL**	O 66	LEU 191	H-donor	3.35	−3.10	−8.36
	O 66	ASN 193	H-donor	2.99	−2.90	
	O 20	GLY 307	H-acceptor	3.12	−1.20	
	O 53	ALA 83	H-acceptor	3.12	−1.10	
**FeL**	CL 66	LEU 189	H-donor	3.68	−0.80	−7.75
	O 67	GLY 306	H-donor	3.01	−3.60	
	O 53	ASN 193	H-acceptor	3.03	−1.00	
**CoL**	O 69	LEU 191	H-donor	3.09	−10.10	−8.15
	O 69	ASN 193	H-donor	3.03	−1.80	
	O 20	ALA 83	H-acceptor	3.19	−1.10	
**NiL**	O 69	LEU 191	H-donor	3.23	−4.30	−8.37
	O 69	ASN 193	H-donor	2.91	−2.70	
	O 20	ALA 83	H-acceptor	3.15	−1.10	
**CuL**	O 66	LEU 191	H-donor	3.20	−4.20	−8.36
	O 66	ASN 193	H-donor	2.92	−2.50	
	O 52	ALA 83	H-acceptor	3.15	−1.00	

## Data Availability

The raw/processed data generated in this work are available upon request from the corresponding author.

## Supplementary Materials

The following supporting information can be downloaded at: https://www.mdpi.com/article/10.3390/ijms232415614/s1.
