# Supplementary material for: Synthesis, DFT, Biological and Molecular Docking Analysis of Novel Manganese(II), Iron(III), Cobalt(II), Nickel(II), and Copper(II) Chelate Complexes Ligated by 1-(4-Nitrophenylazo)-2-naphthol
Source: Int J Mol Sci. 2022 Dec 9;23(24):15614. doi: 10.3390/ijms232415614 (PMC9779307; doi:10.3390/ijms232415614)
Supplement: Supplementary file 1 [file ijms-23-15614-s001.zip › ijms-2032921-supplementary.pdf]

Supporting data

Synthesis, DFT, biological and molecular docking analysis of novel manganese(II), iron(III), cobalt(II), nickel(II), and copper(II) chelate complexes ligated by 1-(4-nitrophenylazo)-2-naphthol

Yousef A. A. Alghuwainem[1], Hany M Abd El-Lateef[2,3*], Mai M Khalaf[2,3], Amer A. Amer[3], Antar A. Abdelhamid[3, 4], Ahmed A. Alzharani[4], Anas Alfarsi[4], Saad Shaaban[2,5*], Mohamed Gouda[1], Aly Abdou[3]

[1]Department of Veterinary public health and care, College of Veterinary Medicine, King Faisal University, Al-Ahsa 31982, Saudi Arabia

[2]Department of Chemistry, College of Science, King Faisal University, Al-Ahsa 31982, Saudi Arabia

[3] Department of Chemistry, Faculty of Science, Sohag University, Sohag 82534, Egypt

[4] Department of chemistry, faculty of science, Albaha University, Albaha, Saudi Arabia

[5] Chemistry Department, Faculty of Science, Mansoura University, Mansoura 35516, Egypt

* Corresponding authors: hmahmed@kfu.edu.sa, hany_shubra@science.sohag.edu.eg (H. M. Abd El-Lateef), Sibrahim@kfu.edu.sa (S. Shaaban).

List of Figures;

Figure S1: 1H-NMR and 13C-NMR of the azo-ligand (HL)

Figure S2: mass spectral of the subject complexes

Figure S3: The UV-vis. Spectra of the free ligand and its complexes.

Figure S4: Determination of composition of the metal complexes by jobs method of continuous variation method

^{1}H-NMR

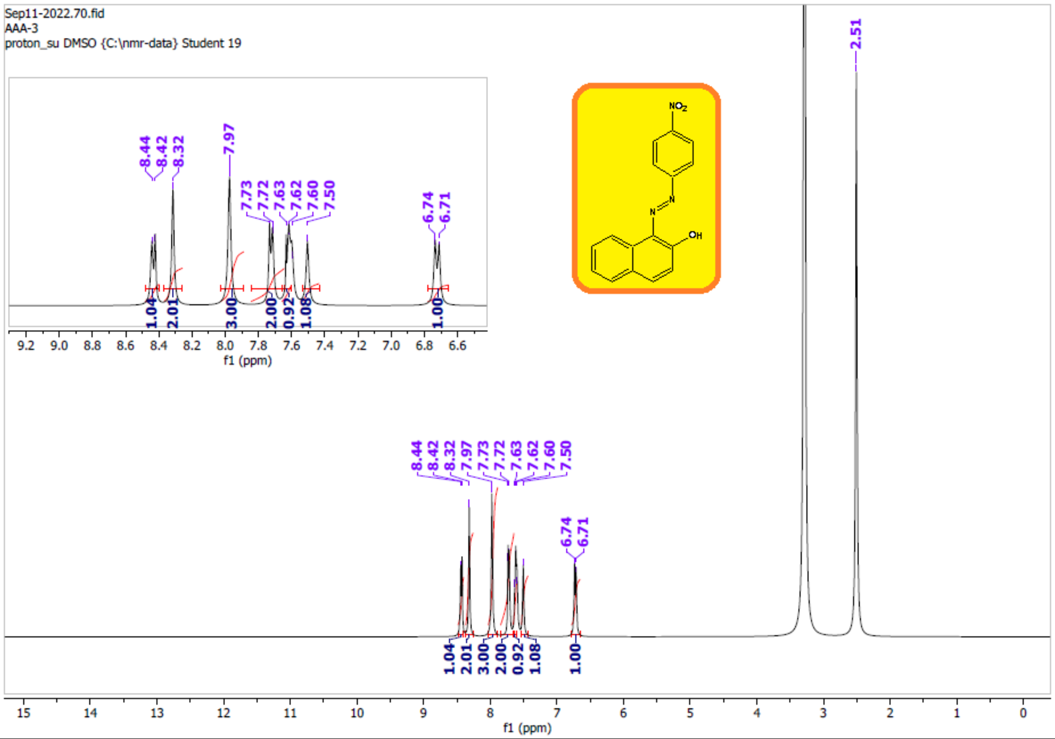

^{13}C-NMR

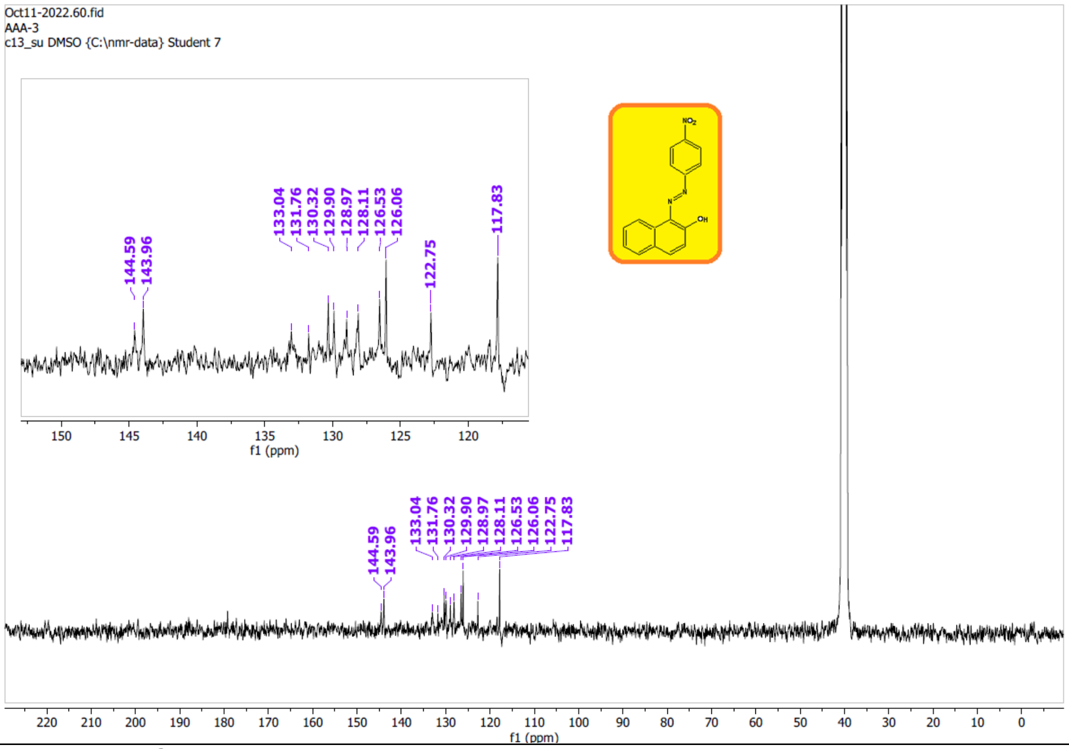

Figure S1: ^{1}H-NMR and ^{13}C-NMR of the azo-ligand (HL)

MnL

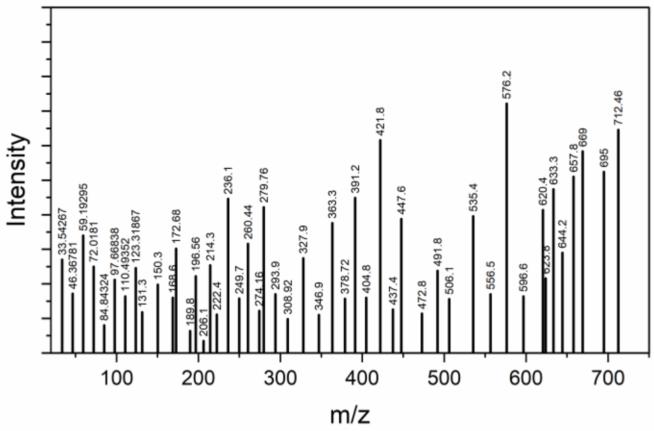

Intensity
m/z
100 200 300 400 500 600 700
33.54267
46.36781
59.19295
72.0181
84.84324
97.66838
110.49352
123.31867
131.3
150.3
168.6
172.68
189.8
196.56
206.1
214.3
222.4
236.1
249.7
260.44
274.16
279.76
293.9
308.92
327.9
346.9
363.3
378.72
391.2
404.8
421.8
437.4
447.6
472.8
491.8
506.1
535.4
556.5
576.2
596.6
620.4
623.8
633.3
644.2
657.8
669
695
712.46

FeL

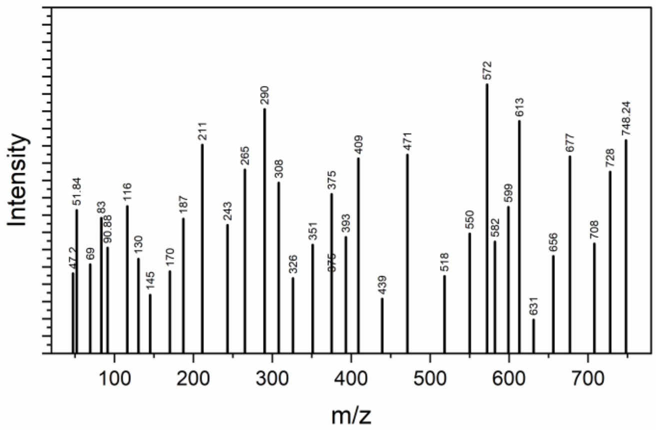

Intensity
m/z
100 200 300 400 500 600 700
47.2
51.84
69
83
90.88
116
130
145
170
187
211
243
265
290
308
326
351
375
376
393
409
439
471
518
550
572
582
599
613
631
656
677
708
728
748.24

CoL

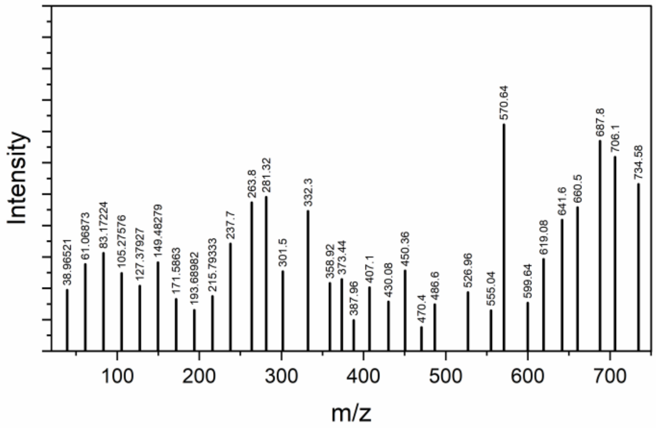

Intensity
m/z
100 200 300 400 500 600 700
38.96521
61.06873
83.17224
105.27576
127.37927
149.48279
171.5863
193.68982
215.79333
237.7
263.8
281.32
301.5
332.3
358.92
373.44
387.96
407.1
430.08
450.36
470.4
486.6
526.96
555.04
570.64
599.64
619.08
641.6
660.5
687.8
706.1
734.58

NiL

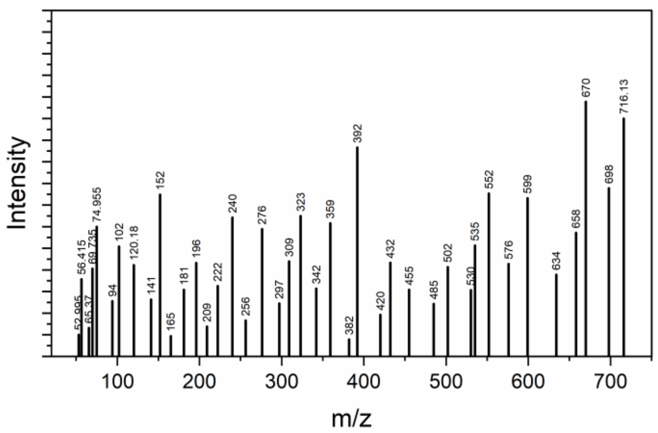

CuL

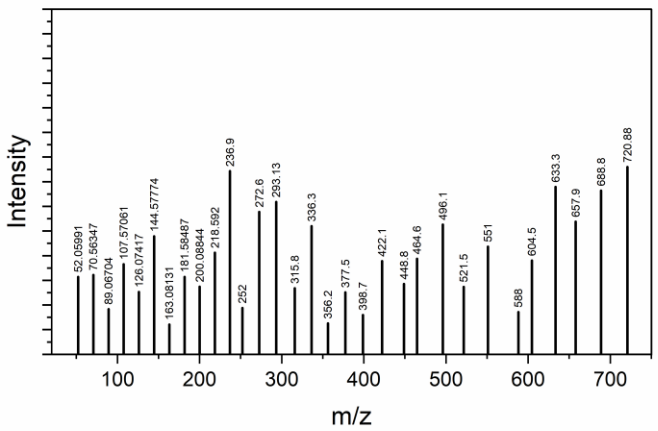

Figure S2: mass spectra of the investigated compounds

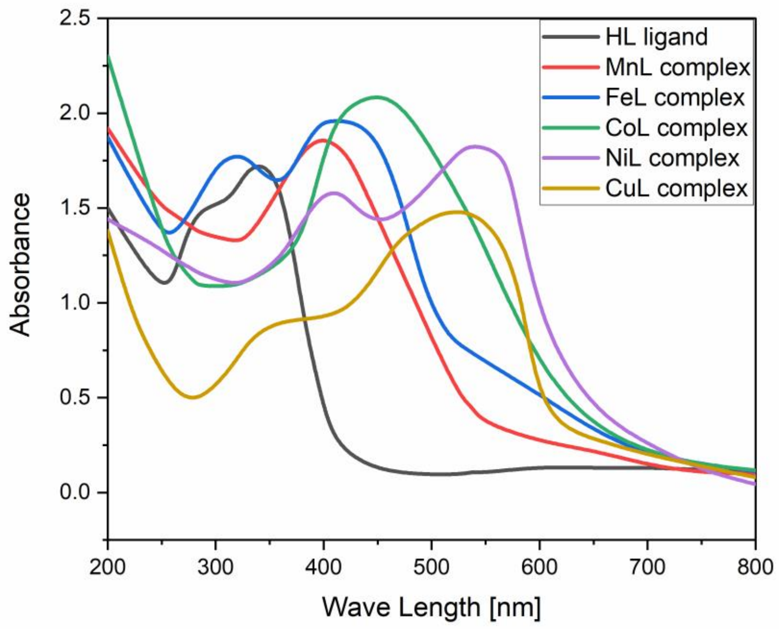

Figure S3: Electronic spectra

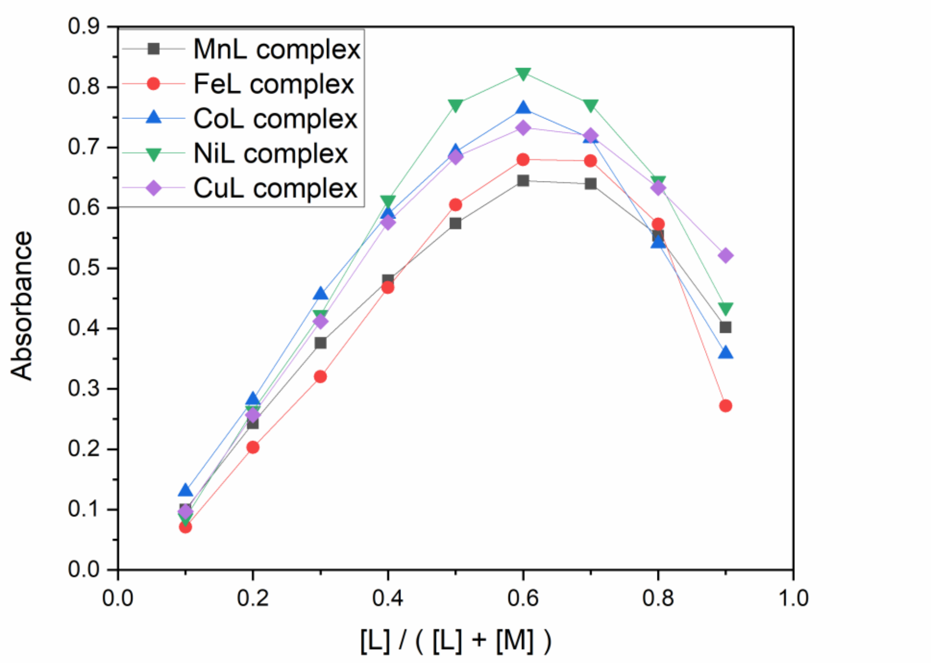

Figure S4: Determination of composition of the metal complexes by continuous variation method

Table S1: Comparison between the minimum inhibition concentration (MIC) of the current compounds with previously reported compounds in the literature survey against e. coli.

	**E. coli (G^-)**	
		**MIC**
1	**MnL[This work]**	**6.25**
2	**FeL[This work]**	**12.5**
3	**CoL[This work]**	**12.5**
4	**NiL[This work]**	**25**
5	**CuL[This work]**	**12.5**
6	**FeL [1]**	50.00
7	**CoL [1]**	55.00
8	**NiL [1]**	60.00
9	**CuL [1]**	50.00
10	**ZnL [1]**	70.00
11	**FeLQ [2]**	25.50
12	**FeLB [2]**	22.50
13	**FeLI [2]**	23.50
14	**CuLQ [3]**	21.50
15	**CuLB [3]**	22.50
16	**CuLI [3]**	22.50
17	**NiLQ [4]**	35.50
18	**NiLB [4]**	34.50
19	**NiLI [4]**	37.50
20	**complex M_5 [36]**	25
21	**[Zn(DIPO)Cl2] [37]**	80
22	**[Co(DIPO)Cl2] [37]**	60
23	**Na[Ni(L1)3] [38]**	< 20
